# Use of prescription opioids in Israel and socio-economic correlations between 2010 and 2020

**DOI:** 10.1186/s13584-024-00598-9

**Published:** 2024-03-07

**Authors:** Limor Adler, Bar Cohen, Shirley Shapiro Ben Daviv, Ori Liran, Daniella Rahamim-Cohen, Afif Nakhleh, Arnon Shahar, Joseph Azuri

**Affiliations:** 1 Health Division, Maccabi Healthcare Services, Tel Aviv, Israel; 2 Department of Family Medicine, Faculty of Medicine, Tel Aviv University, Tel Aviv, Israel; 3 Ben Gurion University, Beer Sheva, Israel; 4 Diabetes and Endocrinology Clinic, Maccabi Healthcare Services, Haifa, Israel; 5 Institute of Endocrinology, Diabetes and Metabolism, Rambam Health Care Campus, Haifa, Israel; 6 The Azrieli Faculty of Medicine, Bar-Ilan University, Safed, Israel

**Keywords:** Opioid usage, Socioeconomic status, Peripherial residency, Arab population, Orthodox-Jews, Minorities

## Abstract

**Background:**

The use of opioids has increased dramatically over the past several years in Israel. The aim of this study was to explore the trends of opioid consumption in Israel over a decade (2010–2020) stratified by socioeconomic status (SES), residence in the periphery, and ethnic background.

**Methods:**

This cross-sectional study included all adult Maccabi Healthcare Services (MHS) patients who filled at least one prescription for opioids during the past decade. In order to standardize dosages and compare different opioid medications, we used the Morphine Milligram Equivalent (MME) conversion factor. We performed The Mann–Kendall test with autocorrelation correction to assess each trend. We then checked the differences between the trends with the Mann–Whitney test (for periphery) and the Kruskal Wallis (for SES and ethnic background).

**Results:**

Between the years 2010–2020, 261,270 MHS members met the study's inclusion criteria. The proportions of opioids consumption were 23.9/1000 patients in 2010 and 27.6/1000 patients in 2020, representing a 15% increase. The average daily consumption of opioids was 4.6 and 10.5 MME in 2010 and 2020, respectively, an increase of 227%. The daily MME during 2020 was higher for residents of the periphery compared to non-periphery residents (daily MME of 14.0 compared to 10.1, respectively). Average daily MME increased gradually during the study period for all levels of SES; the values were highest for the low SES group and the lowest for the high SES group (daily MME in 2020 for the lowest, middle, and high SES groups were 15.2 vs. 11.8 vs. 6.7 respectively).

**Conclusions:**

This study highlights that the primary concern in the increase of opioid use is the increasing dosages. The increase in the number of patients using opioids is also significant but to a minor extent. These phenomena disproportionately impact vulnerable populations. Education programs should be offered to physicians regarding the possible harms of long-term use of opioids. These programs should emphasize the risk factors associated with the development of opioid use disorder (OUD) and the caution needed when increasing dosages or switching to higher-potency drugs. Pain clinics and centers for rehabilitation for patients with chronic pain or OUD should be available, not only in central areas but also in the periphery of the country. These clinics and centers should use a holistic approach and a multidisciplinary team that includes specialists in pain and addiction. They should be financially accessible for patients from low SES group and provide solutions in multiple languages.

**Supplementary Information:**

The online version contains supplementary material available at 10.1186/s13584-024-00598-9.

## Background

Opioids are a class of narcotic analgesic medications used for medium to strong pain alleviation. While opioids are quite efficient in alleviating severe pain in oncological patients and for short-term use in patients after trauma or surgery, they are considered perilous when used chronically due to their addictive nature and the risk of overdose [1].

Over half of the patients prescribed opioids for 90 days will continue their consumption four years later [2]. Evidence of the short-term consequences of opioid use has been demonstrated in many studies, including gastrointestinal symptoms, drowsiness, pruritus and altered mental state [3]. The dangers of chronic opioid use include constipation, stomach aches, tolerance, physiological dependence, withdrawal syndrome, misuse and addiction, hormonal dysregulation, hyper-algesia, cardiovascular ailments (including sudden death), respiratory suppression, and fatal overdose [1].

The use of opioids has increased dramatically over the past several years in the United States of America (USA) and Europe [4–7]. The rise of opioid usage brought about multiple public health and societal consequences on a global scale. In the USA, between 2001 and 2015, cases of opioid intoxication have quadrupled [8]. These alarming trends, and the burden they cause, both financially and socially, have led to significant push-back, with even the president of the USA declaring it an epidemic that has to be addressed urgently [9].

### Opioid use in Israel

In the last decade, several reports showed an increase in the use of all opioids, specifically in Oxycodone combinations and Fentanyl [10–13]. The increase is seen specifically in non-cancer, low SES patients [14]. The aforementioned increase is a compound result of increasing numbers of opioid-using patients, the increase in per-patient dosages, and the duration of use. Another alarming finding is the increase in emergency department visits due to opioid use between 2015 and 2019[15]. According to a 2019 OECD report regarding opioid use in 25 OECD countries, overall, there has been a 20% increase in opioid use between 2011 and 2016, whereas in Israel, a 125% increase was observed [4]. For comparison, other than the use of opioids, Cannabis licensed use has also increased in this time period 10 folds (from 8000 licenses in 2013 to more than 80,000 licenses in 2020) [16]. In 2022, the Ministry of Health (MoH) of Israel instituted an expert board to recommend the necessary steps to cope with the increase in opioid use. One of the recommendations was to enhance monitoring of opioid consumption and start collecting data from all healthcare maintenance organizations (HMOs) in oral morphine equivalent units [17].

This study aimed to explore the trends of opioid consumption in Maccabi Healthcare Services (MHS) throughout a decade (2010–2020) stratified by socioeconomic status, residence in the periphery, and ethnic background.

## Methods

### Study design and setting

This is a cross-sectional study with all MHS patients who filled at least one prescription for an opioid between 2010 and 2020 and were MHS members throughout the entire period. MHS is the second largest HMO in Israel, with more than 2.6 million patients. MHS has electronic medical records (EMR) for all its patients, and all purchases are documented. The study was approved by the ethical committee of MHS (0020-19-MHS). Informed consent was waived due to the study design.

### The study process

We extracted data for 5 commonly used opioids—Fentanyl, Morphine, Oxycodone (including combinations of Oxycodone), Tramadol (including a combination of Tramadol), and Buprenorphine. We did not include Codeine, since it is a weak opioid which is often used as a cough suppressant. In order to standardize the dosages of different agents, we converted all dosages to Morphine Milligram Equivalent (MME). The MME of each opioid reflects its potency relative to morphine. The MME is calculated based on the recommendations of the Centers for Diseases Control and Prevention (CDC); the formula to calculating the MME is multiplying the daily opioid dose for each opioid (the strength per unit * number of units per day) by the conversion factor of the specific opioid [18].

For each patient who purchased opioids during the timeframe of this study, we collected personal data based on their EMR, including birth date, gender, residence in the periphery, socioeconomic status (SES), and ethnic background (Arabs, ultra-orthodox Jews, and the rest of the population). SES levels were defined by the Israeli Central Bureau of Statistics [19]. It was categorized into 3 groups (low [1–4], medium [5, 6], and high [7–10]).

### Statistical analysis

We calculated the average daily consumption in MME, using the formula suggested by the CDC. We assumed that all patients consistently used the medications on the days issued. We repeated the analysis for stratified groups of SES, residency (periphery/non-periphery), and ethnic background (Arabs, ultra-orthodox Jewish, and the rest of the population).

We used the Modified Mann–Kendall test with autocorrelation correction to detect trends during the period of 2010–2020. The Mann–Kendall test is a non-parametric statistical test used to detect trends in data. Since there is an autocorrelation in the data (i.e., patients consumed opioids for successive years), it was essential to correct for it to avoid biased results. Kendall’s Tau was calculated for each series of data, along with the corresponding *p*-value.

In order to check for differences between periphery and non-periphery, the Mann–Whitney test was used, while the Kruskal Wallis test was used to check for differences between the three levels of SES, as well as for the differences between the three groups of ethnic background.

## Results

Between 2010 and 2020, 261,270 MHS members met the inclusion criteria. Of them, almost half (48.2%) were males. One third (36.2%) of patients were 18–40 years old, 40.7% were 41 to 60, and 23.1% were 61 years of age or older. Six percent of patients were Arabs, 4.5% ultra-orthodox Jews, and 89.4% represented the rest of the population. The sample represents all SES groups, with a distribution of 19.6%, 35.4%, and 45% in low SES, middle, and high SES groups, respectively. More than ninety percent of patients (91.6%) were residents of the central area of the country (non-periphery residents).

**Fig. 1:**
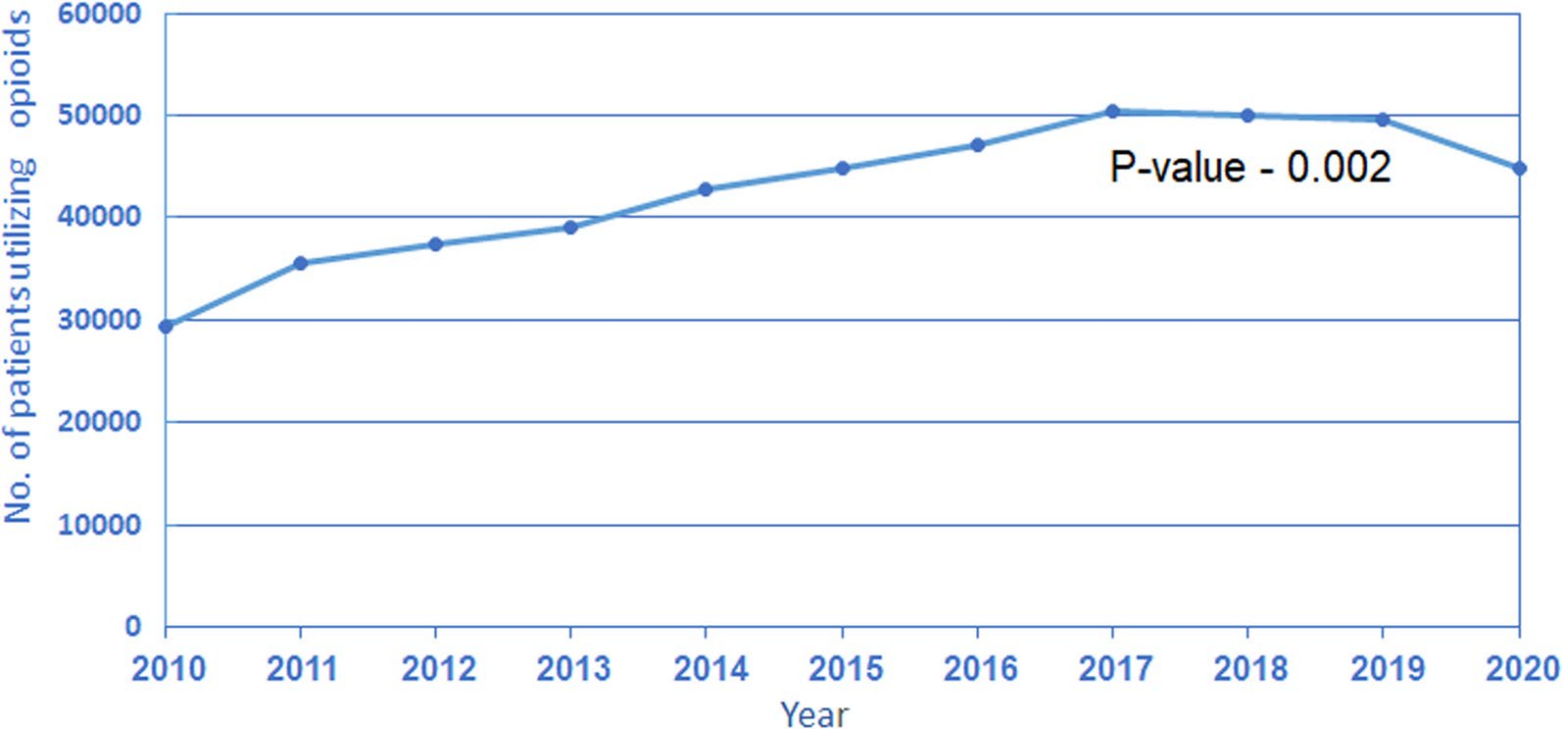
Total number of MHS patients fulfilling at least one prescription of opioids by year

**Fig. 2:**
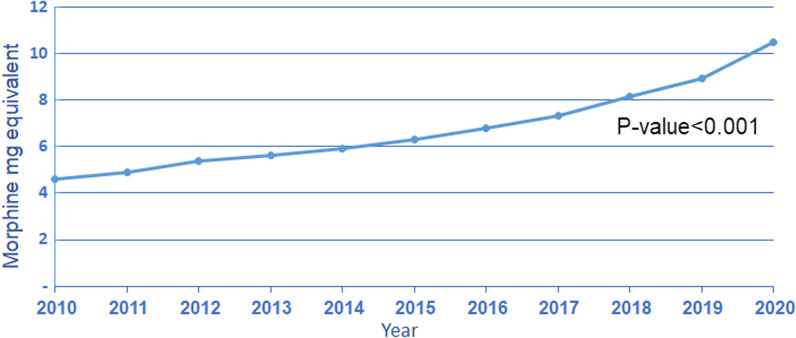
Average MME per patient

The annual number of patients who filled a prescription for opioids increased from 29,367 in 2010 to 44,927 in 2020, Kendall's Tau-0.745, *P*-value-0.002 (Fig. 1). The average daily consumption of opioids per patient who filled prescriptions was 4.6 MME in 2010 as opposed to 10.5 MME in 2020, an increase of 227%, Kendall's Tau-1, *p*-value < 0.001 (Fig. 2). Interestingly, the rate of the increase of MME over time is not uniform but rather accelerating—between 2010 and 2011, the average total MME increased by about 5%; however, this rate increasingly grew, and between 2019 and 2020, there was a 17% increase.

### Usage of opioids stratified by place of residence (periphery/non-periphery)

Throughout the study period, the proportion of patients who resided in the periphery of Israel and filled prescriptions for opioids was greater than patients who did not live in the periphery, *p*-value-0.002 (for example, in 2020, the proportion was 31.0/1000 compared to 27.5/1000, respectively). There was an insignificant increase in both groups; the proportion of patients that live in central regions increased by 15% from 23.9 to 27.6, Kendall's Tau-0.418, *p*-value-0.087. A similar trend was also seen in the periphery (the proportion increased by 6% from 29.4 to 31.0, Kendall's Tau-0.418, *p*-value-0.087) (Additional file 1: Fig. 1S).

**Fig. 3:**
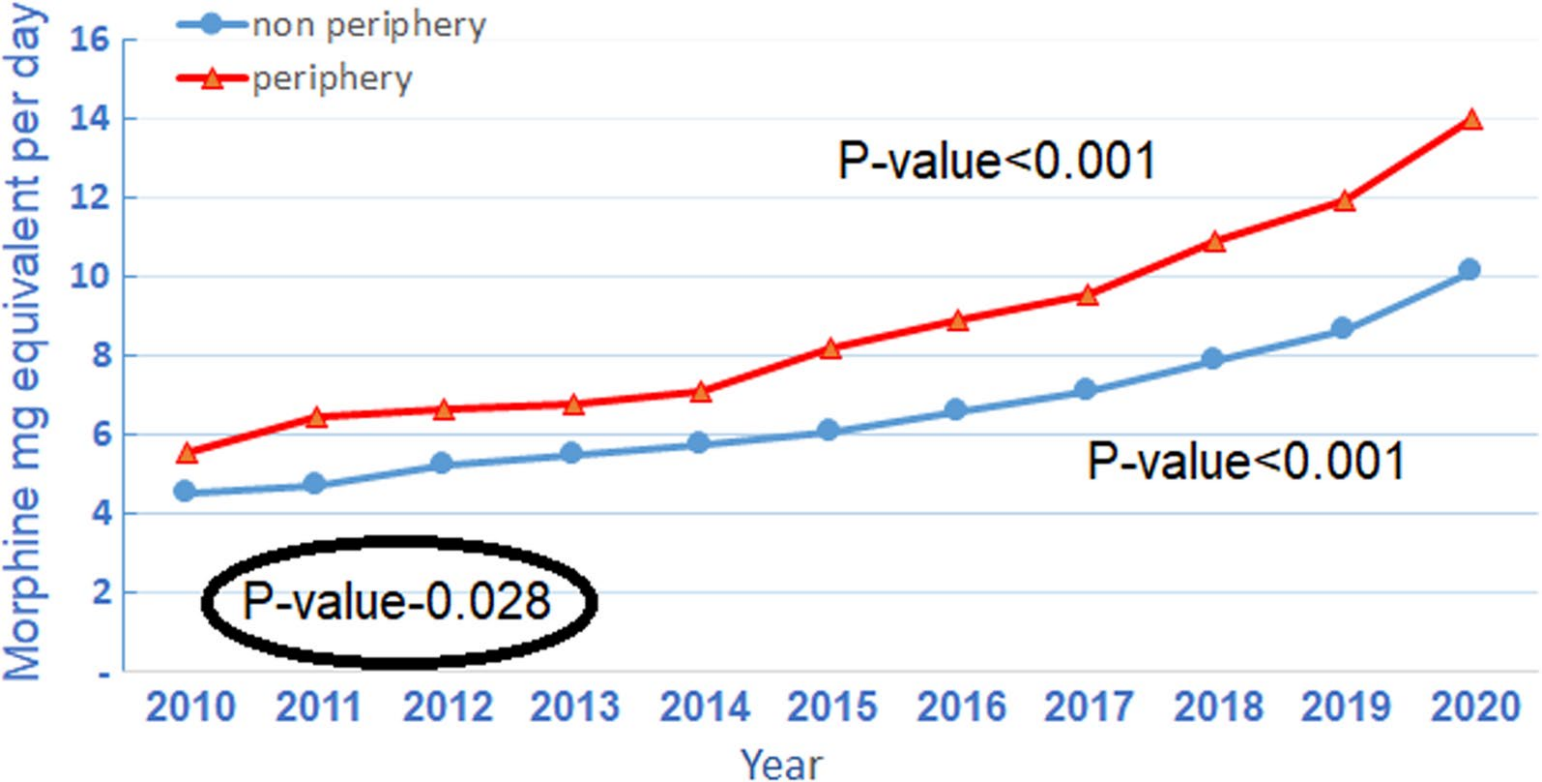
Average daily MME per patient by periphery/non-periphery district and year

Trends in average daily MME were similar in both groups of periphery and non-periphery residents, showing a gradually accelerating increase in MME, Kendall's Tau-1, *p*-value < 0.001 for both (Fig. 3). The corresponding average daily MME per patient among non-periphery residents was 4.5 in 2010 and increased to 10.1 in 2020 (224% increase). The averages for periphery residents increased from 5.6 MME to 14.0 MME (252%). MME was constantly higher for periphery residents throughout the study period, *p*-value-0.028.

### Usage of opioids stratified by socioeconomic status

Analysis by SES showed that throughout the study time frame, patients from lower SES groups filled more prescriptions for opioids than high and medium SES patients, *p*-value-0.002. At the end of the study period, low and medium SES had similar proportions (Additional file 1: Fig. 2S).

**Fig. 4:**
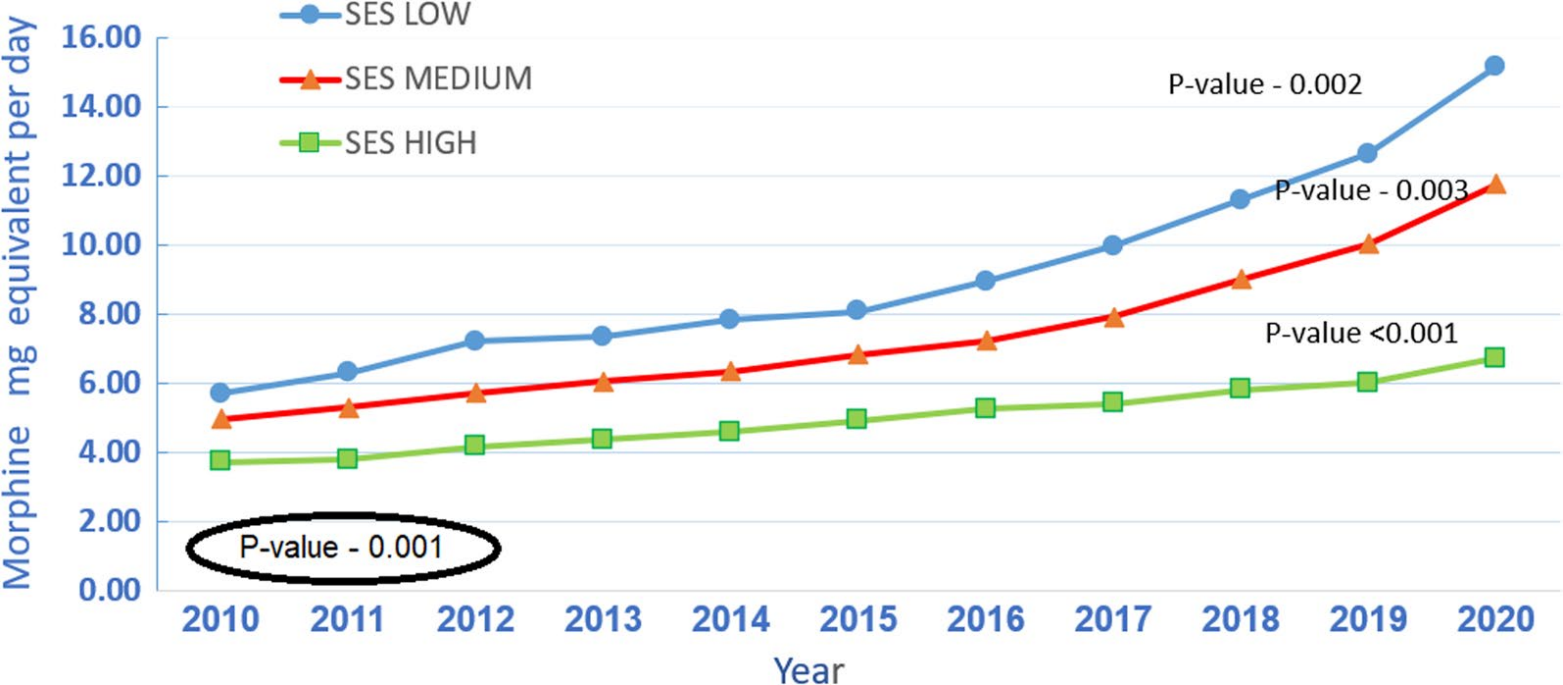
Average daily MME per patient by SES level and year

Average daily MME increased gradually during the 10-year study period for all 3 levels of SES, the values being the highest for the low SES level (Kendall's Tau-1, *p*-value-0.002) and the lowest for the high SES level (Kendall's Tau-1, *p*-value < 0.001), *p*-value-0.001 (Fig. 4).

### Usage of opioids stratified by ethnic background

**Fig. 5:**
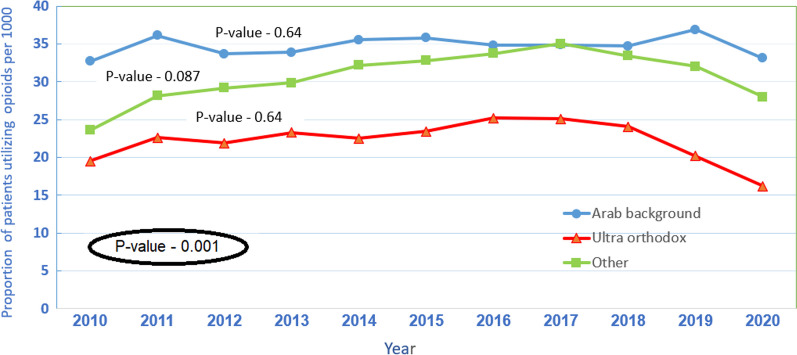
Rate of patients using opioids per 1000 MHS members by population group and year

Throughout the study time frame, Arab patients filled more prescriptions for opioids than ultra-orthodox Jewish and the rest of the population, *p*-value-0.001. The proportion of patients using opioids did not show significant changes; in the Arab population from 32.8/1000 to 33.2/1000, Kendall's Tau-0.127, *p*-value-0.64, in the ultra-orthodox Jewish, from 19.6/1000 to 16.3/1000, Kendall's Tau-0.127, *p*-value-0.64 and in the rest of the population, from 23.7/1000 to 28.1/1000, Kendall's Tau-0.418, *p*-value-0.087 (Fig. 5).

**Fig. 6:**
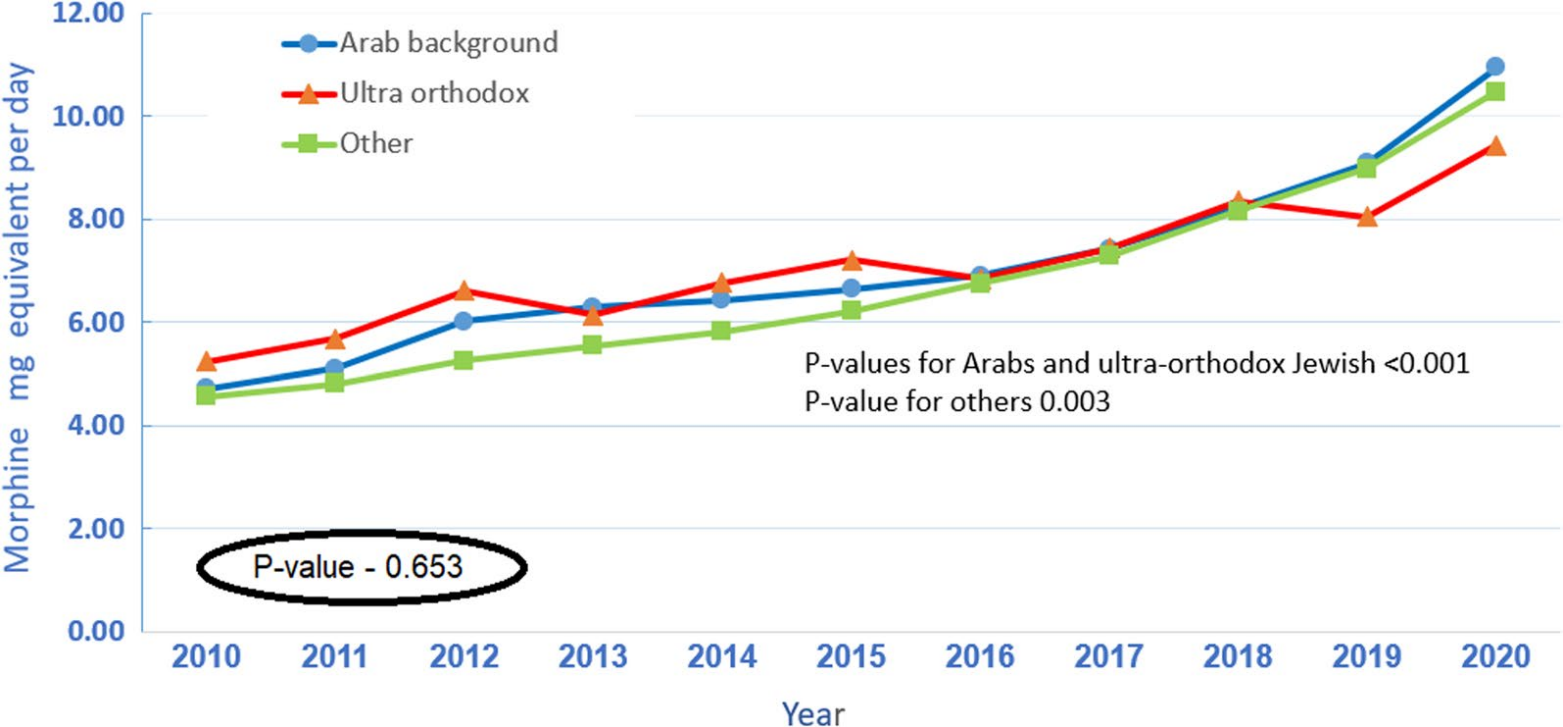
Average daily MME per patient by population group and year

As seen in Fig. 6, average daily MME increased substantially among all ethnic backgrounds; an increase of 240% from 2010 to 2020 among Arabs (from 4.6 to 10.9, Kandell's Tau-1, *p*-value < 0.001), 230% among the the rest of the population (from 4.6 to 10.5, Kandell's Tau-1, *p*-value-0.003), and of 180% among the ultra-orthodox Jewish population (from 5.2 to 9.4, Kandell's Tau-0.891, *p*-value < 0.001). The differences in MME between the groups were not significant, *p*-value-0.653.

## Discussion

### Main findings

This study demonstrated a 15% increase in the proportion of opioid users between 2010 and 2020 (from 23.9/1000 MHS members to 27.6/1000 MHS members) and an increase of 227% in daily MME (from 4.6 in 2010 to 10.5 in 2020). The proportion of opioid usage and the average daily MME in patients residing in the periphery and patients from lower SES groups was higher throughout the study period. The proportion of opioid usage among Arab patients was higher compared to ultra-orthodox Jewish and the rest of the population. However, the average daily MME was similar between Arabs and the rest of the population.

### Interpretation

The increase in the number of patients taking opioids and the increase in MME seen in this study aligns with other studies [6, 7]. This indicates two trends happening concurrently or correlating with one another. The first trend, is the increase in the number of patients taking opioids. This increase plateaued in 2017, possibly due to the increasing awareness globally regarding the potential risks of opioid consumption and the growing discussion in the medical community about it. This plateau is consistent with trends in the United States and Canada [7, 20]. However, this trend is lagged compared to other countries, where a decrease in opioid use has already been reported by this time [7, 20]. While fewer patients filled prescriptions of opioids between 2017 and 2020, the uptick in MME per patient persisted and even displayed an accelerating increase rate. While some of this trend may be attributed to the worsening clinical condition of some of the patients, this can hardly explain the overall continual acceleration in average daily MME throughout the last decade. A possible explanation of the increase in both opioid and medical cannabis use is higher awareness of pain management (by both physicians and patients) and the concept that chronic pain is a disease in itself and not only a symptom [21–23]. Another possibility is the need to try opioid analgesic treatment before a patient with severe pain can receive a medical cannabis license.

The second trend is the increase in MME. The increase in MME may either indicate an increasing dosage of a medication or an up switch to a different opioid with a higher Morphine equivalent (e.g., from Morphine, MME 1 to Fentanyl, MME 2.4). The latter is the more likely explanation in light of the increase in prescriptions for Fentanyl in the country [13]. Misuse of transdermal patches of fentanyl is possible by extracting the material from the patch and administering it intravenously or having it insufflated or inhaled [24]. Due to its potential risks, MHS launched in 2023 a new program meant to decrease opioid use, specifically fentanyl patches. Before the program, fentanyl patches could have been prescribed by any physician. Now, however, a pre-approval by pain specialists is required. This is in order to reduce the use of this potentially harmful medication.

Whether the reason for the increase in MME is an up switch or a dose increase, this trend may be a reflection of known long-term effects of opioids on pain sensitivity and tolerance to opioid agents—that is, over time, for chronic users, increased levels of pain may be present despite opioid use [25]. This basic feature of the opioid class of agents is at the heart of its concerning addiction consequences.

Another noteworthy trend is revealed when comparing the consumption of opioids in the periphery as opposed to the central region in Israel. Here one should note that the periphery and central divide in the country is more than geographic; it is socioeconomic. The periphery in Israel is generally considered to be less wealthy [26, 27]. The higher proportion and MME of opioids in deprived areas are not unique to Israel, though each country has a different intersection of periphery and low SES [28]. A study from the United States reported a significantly higher risk for opioid use and opioid-associated mortality in more deprived areas in the country [29]. This is supported by further studies in the USA [30, 31]. Additionally, the average daily MME in the periphery is consistently higher and presents with an accelerating increase pattern compared to the average dosage in the central region of Israel. One may consider whether this is caused by fewer pain management alternatives, as the periphery in Israel has consistently had scarce healthcare resources, as demonstrated in the MoH’s report on inequality in health [32].

These trends are also observed in different SES groups. Patients from the lowest SES group have the highest proportion of opioid consumption, as seen in other studies from Israel [13, 14]. However, throughout the study period, a consistent increase in the proportions of opioid consumption in patients from the middle SES group was noted and approached those of the lowest SES group by the end of the study period. The increase in MME dosage is seen in all SES groups but is constantly higher in the lowest SES groups. Another worth mentioning finding is the widening gap in MME between low SES compared to high SES (2.6 fold increase compared to 1.8 fold increase, respectively). This is consistent with the trends and widening gap recorded in the periphery of the country, which is, as noted, less wealthy and include higher proportions of low SES patients. This corroborates with findings from the USA [30, 31]. The interaction between SES and periphery seems obvious, but should be further evaluated to understand where intervention is more needed in the periphery or in low SES groups.

The final analysis performed in this study investigated the various ethnic backgrounds comprising Israeli society. The ultra-orthodox Jewish community and the Arab population are considered the two predominant minority groups in Israel representing 12% and 20% of the total population [33]. They share unique characteristics such as large families, low SES, crowded living conditions and a community life [33, 34]. The trends they display vary significantly from that of the rest of the population. In the Arab community, the proportion of patients who filled prescriptions for opioids was considerably higher. In the ultra-orthodox community, the proportion was significantly lower. The MME increase trends were similar in all three groups. It is difficult to put this finding in a global perspective, given the differences in demographic and political nature and the construct of the healthcare system. However, there is evidence suggesting unproportional adverse effects of opioid use in certain ethnic groups in the USA (e.g., African Americans, Hispanics) [35, 36] and higher proportions of opioid prescription among certain populations [37] the Possible explanations are complex. People from lower SES groups and ethnic or religious minorities might rely on more physical or precarious work; they might be more prone to work accidents, predisposing them to opioid exposure and, subsequently, opioid use disorder (OUD) [38]. Economic and social issues are closely associated with opioid use, mainly as a quick solution to complex health problems involving pain, psychological trauma, disadvantages, social isolation, or hopefulness [39].

### Strengths and limitations

This study has several limitations that should be considered. First, MHS patients are not uniformly distributed in Israel, demographically and geographically, suggesting that patients from different ethnic backgrounds, such as Arabs or ultra-orthodox Jewish patients may be underrepresented. Second, we did not look into the reasons patients received opioids in this study. Pain management approaches have changed in the recent years, and dosing and clinical conditions and symptoms to which opioid medication is appropriate has changed. This study did not address the question of why opioids were given to patients, but merely how much opioids were filled by them. Third, we did not include Codeine in this analysis.

This study has several strengths. First, the nationwide coverage and large number of patients. Second, is the use of social determinants of health to identify specific groups in the country that might need specific intervention to reduce opioid consumption.

### Implications

This study highlights that the primary concern in the increase of opioid use is the increasing dosages (as evidenced by the 227% increase in MME). The increase in the number of patients taking opioids is also significant but to a minor extent. Prevention and management of these two phenomena can be done on several levels. Primary prevention is the foremost opportunity to stabilize the rate of prescriptions. This can be done by continuously educating physicians about the possible harms of opioids along with the appropriate conditions and symptoms to which opioids may be prescribed. The CDC guidelines recommend not to use opioid pharmacotherapy for chronic pain as first-line therapy [18]. It is important to provide tools to physicians to identify those at risk to develop OUD. Such tools can be implanted in the EMR and screen for those patients at increased risk for OUD and alert the physician before the first prescription is handed. Secondary prevention is possible by avoiding increasing the dosages of patients already on opioids and trying to avoid switching to higher-potency drugs when it is not appropriate. This can be done through education programs for physicians, by monitoring opioid prescriptions and by requiring approval from a pain specialist for certain opioids, like the decision made by MHS regarding Fentanyl in 2023. The CDC guidelines recommend to discuss the risk and benefits of opioids before starting and periodically during the treatment [18]. Consulting pain specialists in designated multidisciplinary clinics, when patients ask for higher dosages of opioids, may result in other therapeutic options. Tertiary prevention should be aimed at patients already receiving high dosages of opioids or having an OUD. The CDC guidelines recommend to offer treatment for OUD when it is identified in patients [18]. Centers for rehabilitation should be available, not only in central areas but also in the periphery of the country. They should be financially accessible through all HMOs for patients from low SES backgrounds and provide solutions in multiple languages. Medications for treating OUD should be included in the basic health basket and offered to patients when relevant.

## Conclusions

In this study, we explored opioid use trends in Israel between 2010 and 2020. We found an overall 15% increase in the proportion of patients taking opioids and an increase of 227% in the daily average MME. Throughout the entire study timeframe, the proportion of opioid prescription was higher for patients from the periphery of the country, patients from the lowest SES group, and the Arab population. Average daily MME was consistently higher for patients from the periphery of Israel and patients from the lowest and middle SES groups.

The results found here reflect the dangerous nature of this class of agents and its unproportioned effect on more vulnerable patients. The increase in MME per patient was much more substantial than the increase in the proportion of patients filling prescriptions. This is meaningful on a personal and public health level, given the long-term consequences and costs of OUD, which have been reported in other countries. We suggest that the Israeli MoH prepare intervention plans for these populations at the highest risk for use, low SES, Arab background and patients living in the periphery of the country.

### Supplementary Information

**Additional file 1: Fig. 1S**. Rate of patients using opioids per 1000 MHS members by periphery/non-periphery district and year, 2010-2020.

**Additional file 2: Fig. 2S**. Rate of patients using opioids per 1000 MHS members by SES and year, 2010-2020.

## Data Availability

The datasets generated and/or analyzed during the current study are not publicly available due to ethical restrictions.

## Abbreviations

HMO: Healthcare Maintenance organization
MHS: Maccabi Healthcare Services
MME: Morphine Milligram Equivalent
OUD: Opioid Use Disorder
SES: Socioeconomic status
USA: United States of America
