# Supplementary figures and images for: Use of prescription opioids in Israel and socio-economic correlations between 2010 and 2020
Source: Isr J Health Policy Res. 2024 Mar 7;13:12. doi: 10.1186/s13584-024-00598-9 (PMC10918956; doi:10.1186/s13584-024-00598-9)

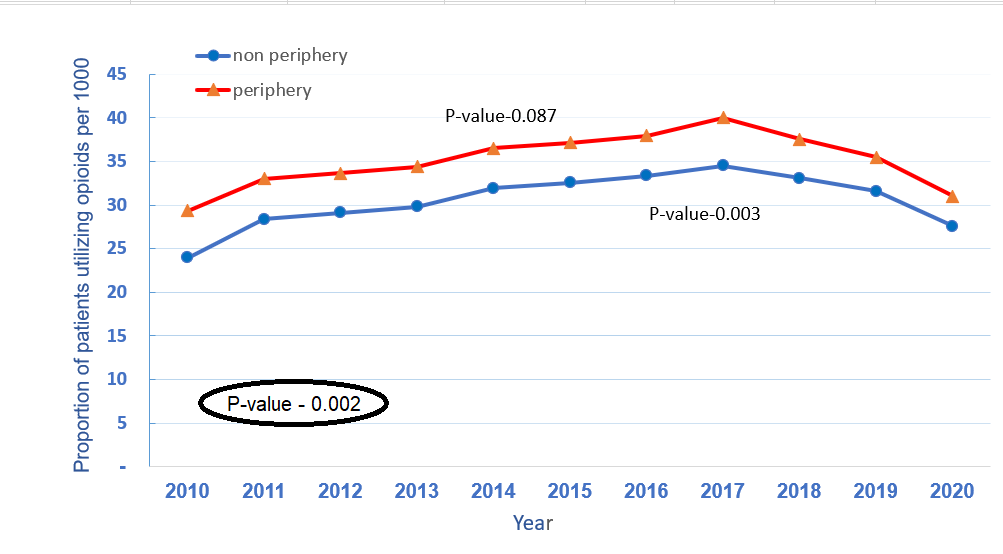

Supplement: Supplementary file 1 — Additional file 1: Fig. 1S. Rate of patients using opioids per 1000 MHS members by periphery/non-periphery district and year, 2010-2020. [file 13584_2024_598_MOESM1_ESM.png]

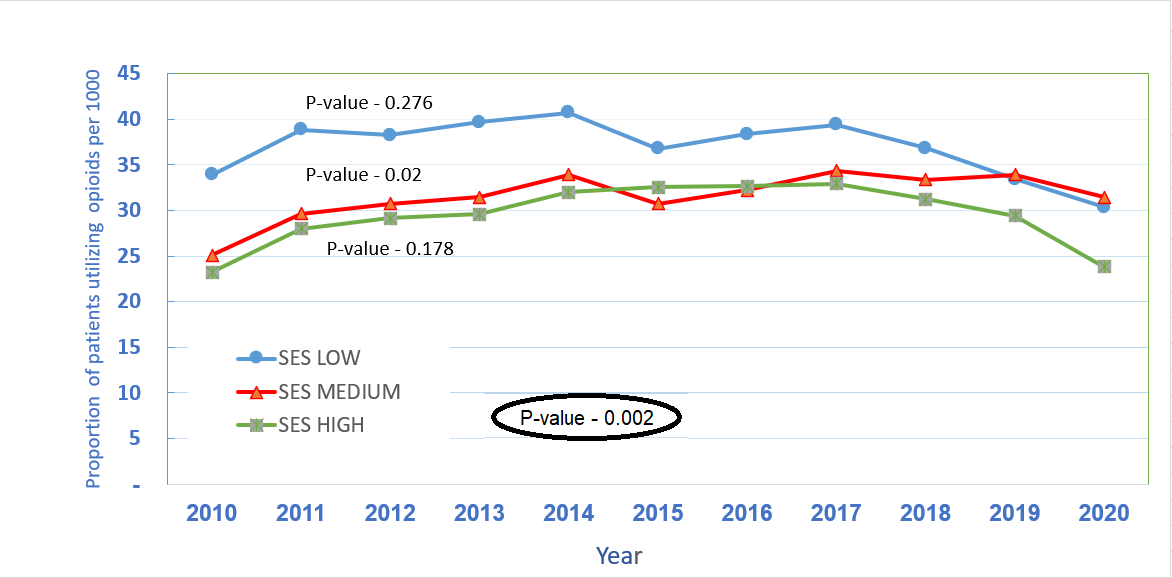

Supplement: Supplementary file 2 — Additional file 2: Fig. 2S. Rate of patients using opioids per 1000 MHS members by SES and year, 2010-2020. [file 13584_2024_598_MOESM2_ESM.png]
